# Tailoring grain sizes of the biodegradable iron-based alloys by pre-additive manufacturing microalloying

**DOI:** 10.1038/s41598-021-89022-9

**Published:** 2021-05-05

**Authors:** Chih-Chieh Huang, Tu-Ngoc Lam, Lia Amalia, Kuan-Hung Chen, Kuo-Yi Yang, M. Rifai Muslih, Sudhanshu Shekhar Singh, Pei-I. Tsai, Yuan-Tzu Lee, Jayant Jain, Soo Yeol Lee, Hong-Jen Lai, Wei-Chin Huang, San-Yuan Chen, E-Wen Huang

**Affiliations:** 1 Department of Materials Science and Engineering, National Yang Ming Chiao Tung University, Hsinchu, 30013 Taiwan; 2 Department of Physics, College of Education, Can Tho University, Can Tho City, 900000 Vietnam; 3 Teknik Material dan Metalurgi, Institut Teknologi Kalimantan, Balikpapan, 76127 Indonesia; 4 Biomedical Technology and Device Research Laboratories, Industrial Technology Research Institute, Hsinchu, 310 Taiwan; 5 Neutron Scattering Lab. PSTBM-BATAN, Kawasan PUSPIPTEK Serpong, 15314 Indonesia; 6 Department of Materials Science and Engineering, Indian Institute of Technology Kanpur, Kanpur, UP 208016 India; 7 Department of Materials Science and Engineering, National Taiwan University, Taipei, 10607 Taiwan; 8 Department of Materials Science and Engineering, Indian Institute of Technology, New Delhi, 110016 India; 9 Department of Materials Science and Engineering, Chungnam National University, Daejeon, 34134 Republic of Korea; 10 Material and Chemical Research Laboratories, Industrial Technology Research Institute, Hsinchu, 310 Taiwan; 11 Laser and Additive Manufacturing Technology Center, Industrial Technology Research Institute, Hsinchu, 31040 Taiwan

**Keywords:** Mechanical properties, Metals and alloys

## Abstract

We demonstrated the design of pre-additive manufacturing microalloying elements in tuning the microstructure of iron (Fe)-based alloys for their tunable mechanical properties. We tailored the microalloying stoichiometry of the feedstock to control the grain sizes of the metallic alloy systems. Two specific microalloying stoichiometries were reported, namely biodegradable iron powder with 99.5% purity (BDFe) and that with 98.5% (BDFe-Mo). Compared with the BDFe, the BDFe-Mo powder was found to have lower coefficient of thermal expansion (CTE) value and better oxidation resistance during consecutive heating and cooling cycles. The selective laser melting (SLM)-built BDFe-Mo exhibited high ultimate tensile strength (UTS) of 1200 MPa and fair elongation of 13.5%, while the SLM-built BDFe alloy revealed a much lower UTS of 495 MPa and a relatively better elongation of 17.5%, indicating the strength enhancement compared with the other biodegradable systems. Such an enhanced mechanical behavior in the BDFe-Mo was assigned to the dominant mechanism of ferrite grain refinement coupled with precipitate strengthening. Our findings suggest the tunability of outstanding strength-ductility combination by tailoring the pre-additive manufacturing microalloying elements with their proper concentrations.

## Introduction

In August 2020, Gartner Research reported that biodegradable sensors are one of the five emerging technologies^1^. The first biodegradable application is for the suture with polymers used as the key material^2,3^. For the biodegradable implants, the three major metallic materials are the magnesium (Mg)^4^, zinc (Zn)^5,6^, and iron (Fe)^7,8^ alloys. The earliest development of the biodegradable materials was Mg-based alloy which has good biocompatibility in human body. However, due to its relatively low strength, exploring and developing the alternative biodegradable alloys are requisite^9,10^.

Among those three common metallic systems, the Fe alloys are known to possess the highest mechanical strength for the expectedly tunable mechanical properties and easier to manufacture by various fabrication processes. One of the recently developed application of Fe-based alloys in biomedical field is the biodegradable coronary stent (pure Fe) and biodegradable bone replacement implant (Fe0.6P) which have been identified causing no local or systemic toxicity^11,12^. In general, the coronary stents show unclear function six months after implantation, which makes the development of better biodegradable iron stent rational^13^. For the bone healing applications, biodegradable bone implants were developed to avoid the need of secondary surgery to remove the implants^14^. However, the main issue of earlier observed biodegradable iron coronary stent results from the low degradation rate^15^. The development of biodegradable materials that have high mechanical properties with a suitable degradable rate is imperative for implant applications^16^. It has been reported that the reduced grain size in the biodegradable iron coronary stent would increase the corrosion rate^17^.

Meanwhile, additive manufacturing is a fabrication process where the parts are built layer by layer with a very localized heating from a laser source^18–21^. With the assistance of rapid development of the additive manufacturing, the Fe-based biodegradable implants have drawn much attention because of great possibilities in building complex geometry and customized parts^22,23^. Tailoring the enhanced mechanical properties of pure Fe via grain refinement by the selective laser melting (SLM) process was explored^24,25^. In fact, there have been many effective grain refinement methods reported for the Fe-based alloys and the steels^26–28^. However, achieving grain refinement by undercooling is full of challenges due to recalescence effect during phase transformation and it is even more difficult for a large batch process^29,30^. Up to date, the post heat treatments on the additive manufacturing parts are well summarized^31^.

Microalloying element molybdenum (Mo) has been effectively used to increase the strength of steel and its potential applications as artificial implants have recently been explored^32,33^. Our primary objective is to tailor the microstructure in simultaneously improving both the mechanical properties and degradation rate of the Fe-based alloys by the addition of trace amounts of biodegradable Mo. In the present study, we investigated the design of microalloying elements for tunable mechanical properties via controllable grain sizes in two horizontally built SLM biodegradable Fe-based alloys of BDFe and BDFe-Mo. To understand the dominant mechanisms governing the mechanical properties in the two SLM-built BDFe samples, we carried out monotonic tension experiments. Since thermal stability of the feedstock materials is especially important during the building process^34,35^, the quality of pre-additive manufacturing microalloying BDFe and BDFe-Mo powders during multiple heating and cooling sequences is one of the prerequisites to be examined in this study.

## Results

### Thermal stability during continuous heating

**Figure 1:**
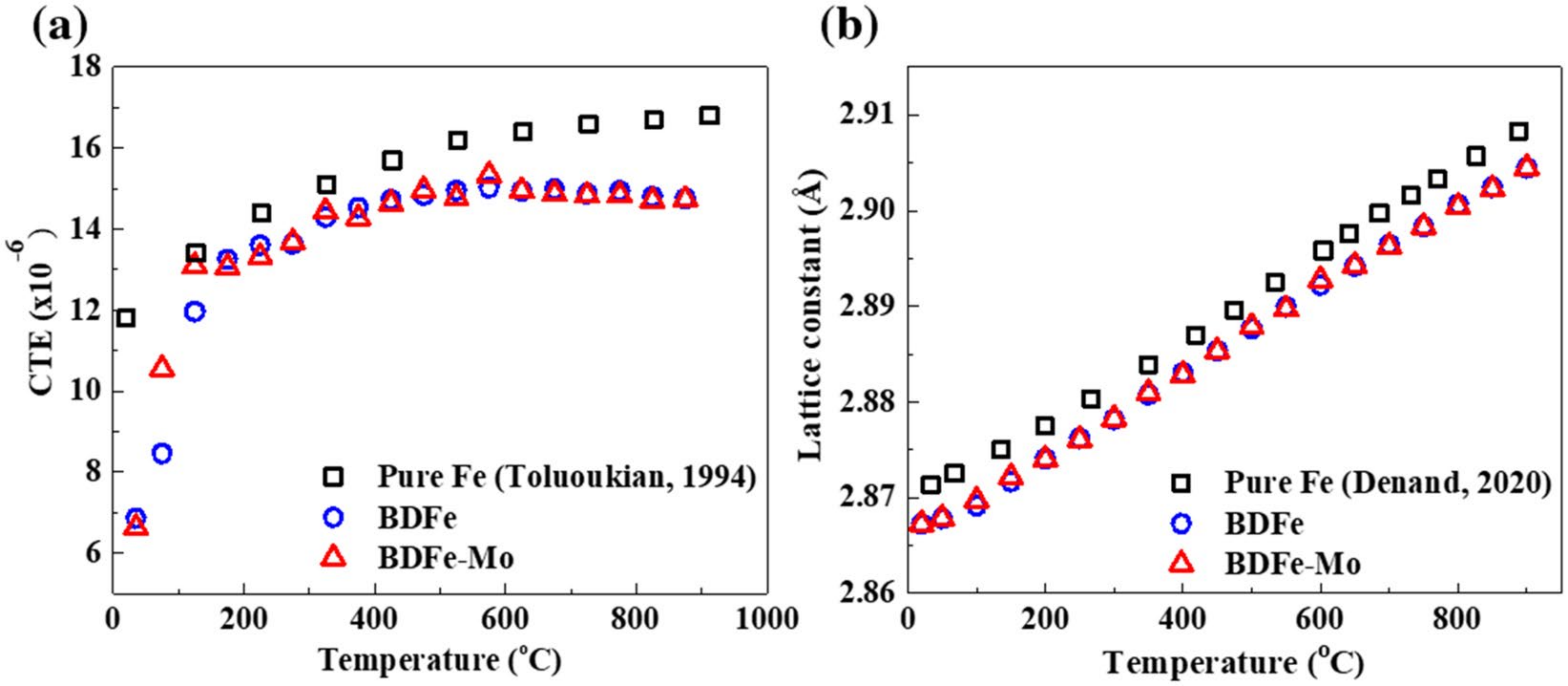
(**a**) Average linear coefficient of thermal expansion and (**b**) lattice constant change upon continuous heating from RT to 900 °C in the two BDFe powders compared with the earlier works of pure Fe^36,43^.

Figure 1 shows the coefficient of thermal expansion (CTE) and lattice constant change using temporally coherent X-ray diffraction upon continuous heating from room temperature (RT) to 900 °C in the two BDFe powders in comparison with the previously reported pure Fe. Two BDFe powders disclosed similar CTE values in Fig. 1a, except those in the temperature range from 50 to 100 °C in which the CTE value of BDFe-Mo was higher than that of BDFe. Moreover, compared with the pure Fe^36^, the two BDFe powders showed lower CTE values. The increased chromium (Cr) and silicone (Si) concentrations were reported to reduce the CTE of the ferritic iron^37^ while the increased carbon (C) and manganese (Mn) contents were found to increase the CTE of the Fe alloys^38–40^. The latter one is supposed to be the dominant contribution to the slightly higher CTE value of the BDFe-Mo although there exist complex competitive interactions between the individual microalloying elements. A slight decrease in the CTE values of both BDFe powders from 500 to 900 °C relates to the transformation into Fe’s magnetic phase from 570 to 877 °C^41,42^. As shown in Fig. 1b, the lattice constants upon continuous heating from RT to 900 °C were similar in the two BDFe powders and they were lower compared with the reported lattice constant of pure Fe^43^, which was in accordance with the variation trend of CTE.

Thermal stability of pre-additive manufacturing powders is very important for a better quality of the as-built parts fabricated via additive manufacturing^20^. Armentani et al*.* reported that a butt welding with higher CTE tends to produce higher residual stresses^44^, which may cause serious limitation on the practical use of the additive manufactured parts due to their influence on plastic deformations, microcracks, and load resistance^45,46^. In addition, material with lower CTE was found to possess even lower CTE value after SLM process^47^. Therefore, the pre-additive manufacturing BDFe powders with lower CTE are expected to enhance the thermal stability and possess good mechanical properties in the SLM-built parts.

### Phase and microstructure stability during thermal cycling

**Figure 2:**
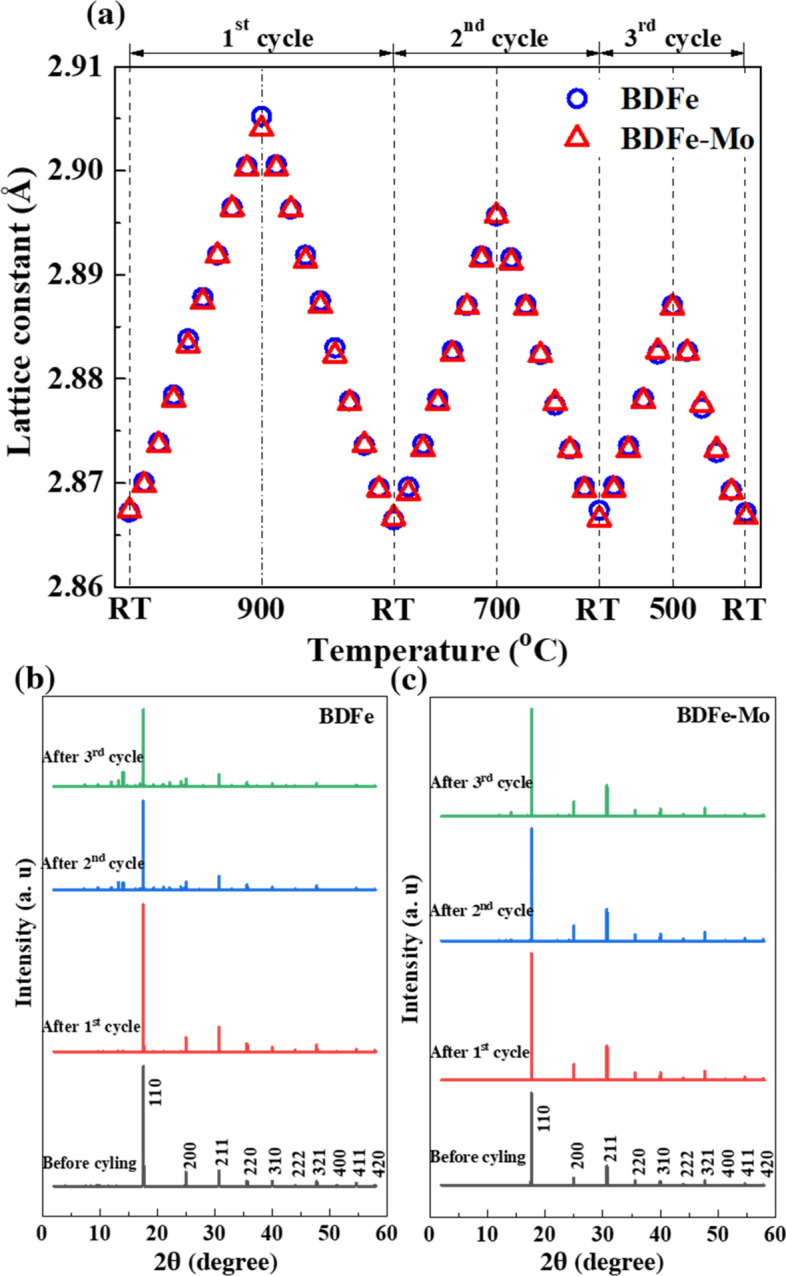
(**a**) Lattice constant change during multiple heating and cooling sequences. The diffraction profiles at RT as a function of thermal cycle in the (**b**) BDFe and (**c**) BDFe-Mo powders.

Thermal cycling test was conducted to identify the phase stability of two BDFe powders, which would be experienced during the SLM process. Figure 2a shows the variation of lattice constant as a function of temperature during the repeated heating and cooling cycles in which no evident difference was obtained between the two BDFe powders. To further acquire the phase transformation during multiple heating and cooling sequences, the evolution of diffraction patterns at RT after each thermal cycle was recorded in Fig. 2b and c. The diffraction profiles of two BDFe powders exhibited a fully body-centered cubic (bcc) Fe with the major diffraction peaks of {110}, {200}, {211}, {220}, {310}, {222}, {321}, {400}, {411}, and {420} before thermal cycling test. However, an appearance of minor peaks was visible after the 2nd cycle and more evident after the 3rd cycle in both BDFe powders, which was assigned to the oxidized hematite (Fe_2_O_3_) and magnetite (Fe_3_O_4_) phases. In addition, more minor peaks were obtained in the BDFe rather than in the BDFe-Mo.

**Figure 3:**
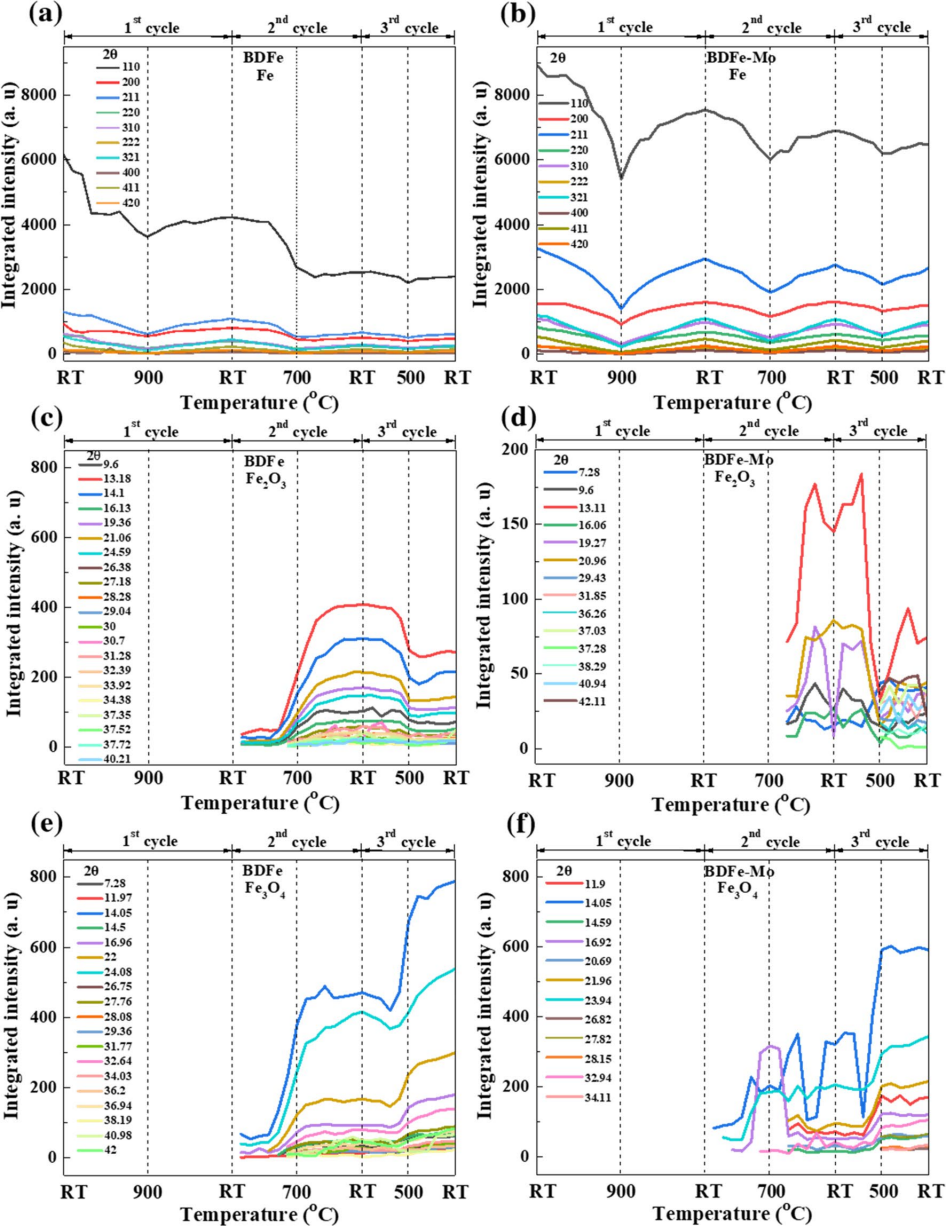
The integrated intensity evolutions of bcc Fe in the (**a**) BDFe and (**b**) BDFe-Mo. Those of Fe_2_O_3_ in the (**c**) BDFe and (**d**) BDFe-Mo. Those of Fe_3_O_4_ in the (**e**) BDFe and (**f**) BDFe-Mo during thermal cycling.

We analyzed the integrated intensities of the major peaks of bcc Fe, the minor peaks of Fe_2_O_3_ and Fe_3_O_4_ to investigate their evolutions during the heating and cooling processes, depicted in Fig. 3. As shown in Fig. 3a and b, both BDFe powders disclosed a similar tendency of decreasing bcc Fe during thermal cycling. In contrast to the reduction of the bcc, the oxidized Fe_2_O_3_ and Fe_3_O_4_ phases increase during the thermal cycling in both BDFe powders. The integrated intensities of Fe_2_O_3_ reached the highest values after the 2nd cycle and then decreased after the 3^rd^ cycle in Fig. 3c, while those of Fe_3_O_4_ in the BDFe continued increasing and got the maximum values after the 3rd cycle in Fig. 3e. The integrated intensity evolutions of Fe_2_O_3_ (Fig. 3d) and Fe_3_O_4_ (Fig. 3f) in the BDFe-Mo was somewhat similar to those in the BDFe.

It is noted that the BDFe-Mo exhibited higher integrated intensity for the bcc Fe but lower intensities for Fe_2_O_3_ and Fe_3_O_4_ phases, as compared to the BDFe. To elucidate the development of bcc Fe, Fe_2_O_3_ and Fe_3_O_4_ phases during the successive heating and cooling sequences, we quantified the phase fraction by calculating their integrated area fractions, as listed in Table 1.

**Table 1:** Phase fraction of bcc Fe, Fe_2_O_3_, and Fe_3_O_4_ at RT during thermal cycling.

Phase	BDFe				BDFe-Mo			
	Before cycling (%)	After 1st cycle (%)	After 2nd cycle (%)	After 3rd cycle (%)	Before cycling (%)	After 1st cycle (%)	After 2nd cycle (%)	After 3rd cycle (%)
Fe	100.0	96.0	46.70	43.20	100.0	96.1	83.40	79.50
Fe_2_O_3_	0.0	0.0	30.15	18.70	0.0	0.0	7.60	0.00
Fe_3_O_4_	0.0	4.0	23.15	38.10	0.0	3.9	9.00	20.50

It can be seen from Table 1 that the amounts of oxidized Fe_2_O_3_ and Fe_3_O_4_ in the BDFe were much higher than those in the BDFe-Mo during thermal cycling, indicating better oxidation resistance of the BDFe-Mo powder. The existence of Mo and the increased Si and Cr contents might hinder the diffusion of Fe cations towards the metal/oxide interface and thus significantly reduce the high temperature oxidation in the BDFe-Mo^48^. The tendency that transforms to the more stable Fe_3_O_4_ phase was also found^49^.

### Tunable mechanical performance

**Figure 4:**
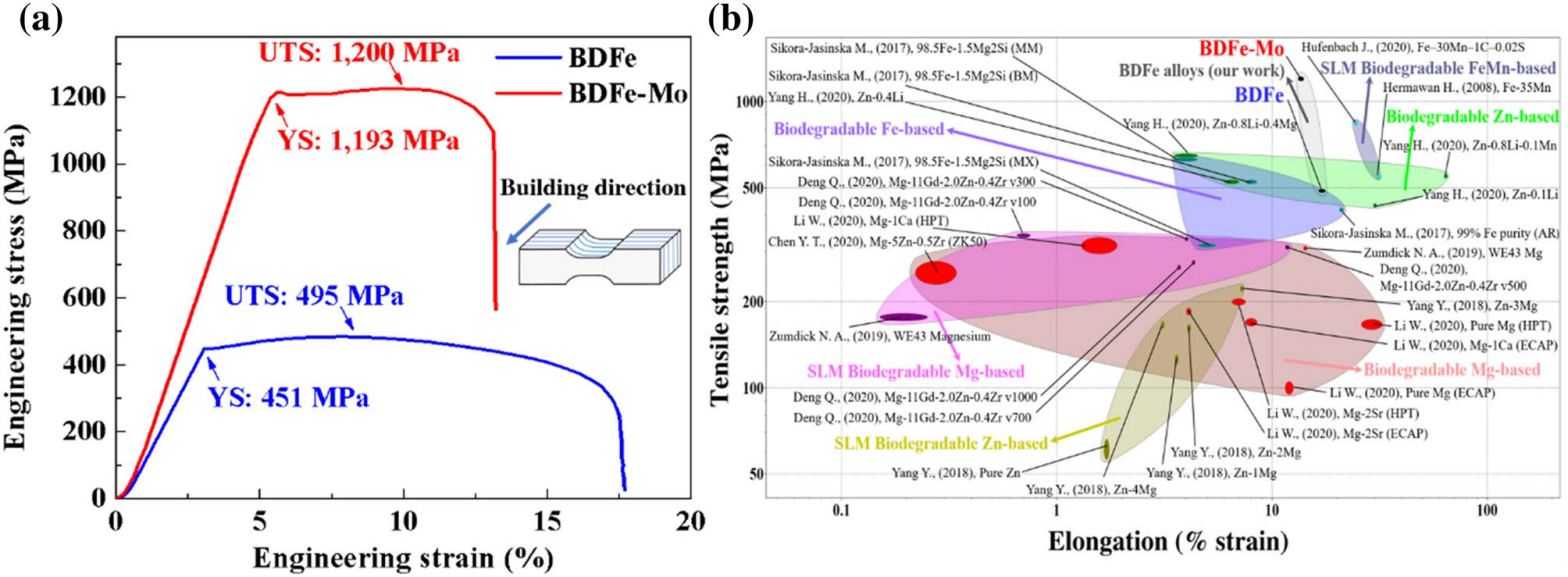
(**a**) Engineering S–S curves of the two BDFe samples and (**b**) tensile strength versus elongation of the two BDFe compared with the Mg-based^50,51,53^, Zn-based^5,56,57^, and Fe-based^52,54,55^ biodegradable alloys. The schematic illustration of horizontally built SLM BDFe specimens was shown in the inset of (**a**).

Figure 4 presents the tensile properties of the two horizontally built SLM BDFe alloys in comparison with other biodegradable alloys. It can be seen from the uniaxial tensile engineering stress strain (S–S) curves in Fig. 4a that a salient strength enhancement was evidently obtained in the BDFe-Mo alloy. Specifically, the macroscopic yield strength (YS) and ultimate tensile strength (UTS) of the BDFe-Mo was 1193 and 1200 MPa, which were 2.5 times greater than those of the BDFe (451 and 495 MPa, respectively). The elongation to fracture was reduced by 23% from the BDFe (17.5%) to the BDFe-Mo (13.5%). Figure 4b depicts the tensile strength and elongation of the two BDFe alloys compared with the Fe, Zn, and Mg-based biodegradable alloys fabricated using the conventional methods and SLM^5,50–57^. Both the SLM-built BDFe alloys revealed superior tensile properties with an especially remarkable mechanical strength observed in the BDFe-Mo. Several possible reasons governing the strengthening mechanism in the BDFe-Mo might be the grain boundary strengthening, the increased dislocation density, and the precipitation of second phase^24^, which will be unraveled for the design of pre-additive manufacturing microalloying elements regarding to the improved strength-ductility combination.

## Discussion

### Grain boundary strengthening effect

One of the general reasons leading to the grain boundary strengthening in laser process manufacturing is the high speed of laser scanning or fast cooling rate. During the SLM process, pure Fe experiences multiple ferrite (α) → austenite (γ) → ferrite (α) phase transformation during the successive cycles of rapid heating and cooling, resulting in hierarchical microstructure with 0.1–0.3 μm sized subgrains, separated by low-angle grain boundaries^25,58^. The optimal laser power of 150 W and faster laser scanning speed of 1.2 m/s were expected to significantly promote the increased nucleation rate and to achieve microstructure refinement of the SLM-built BDFe alloys during rapid solidification process. Although the two BDFe samples experienced the same SLM process, a more significant strengthening effect was attained in the BDFe-Mo, generally originated from the major difference in microstructural evolution.

**Figure 5:**
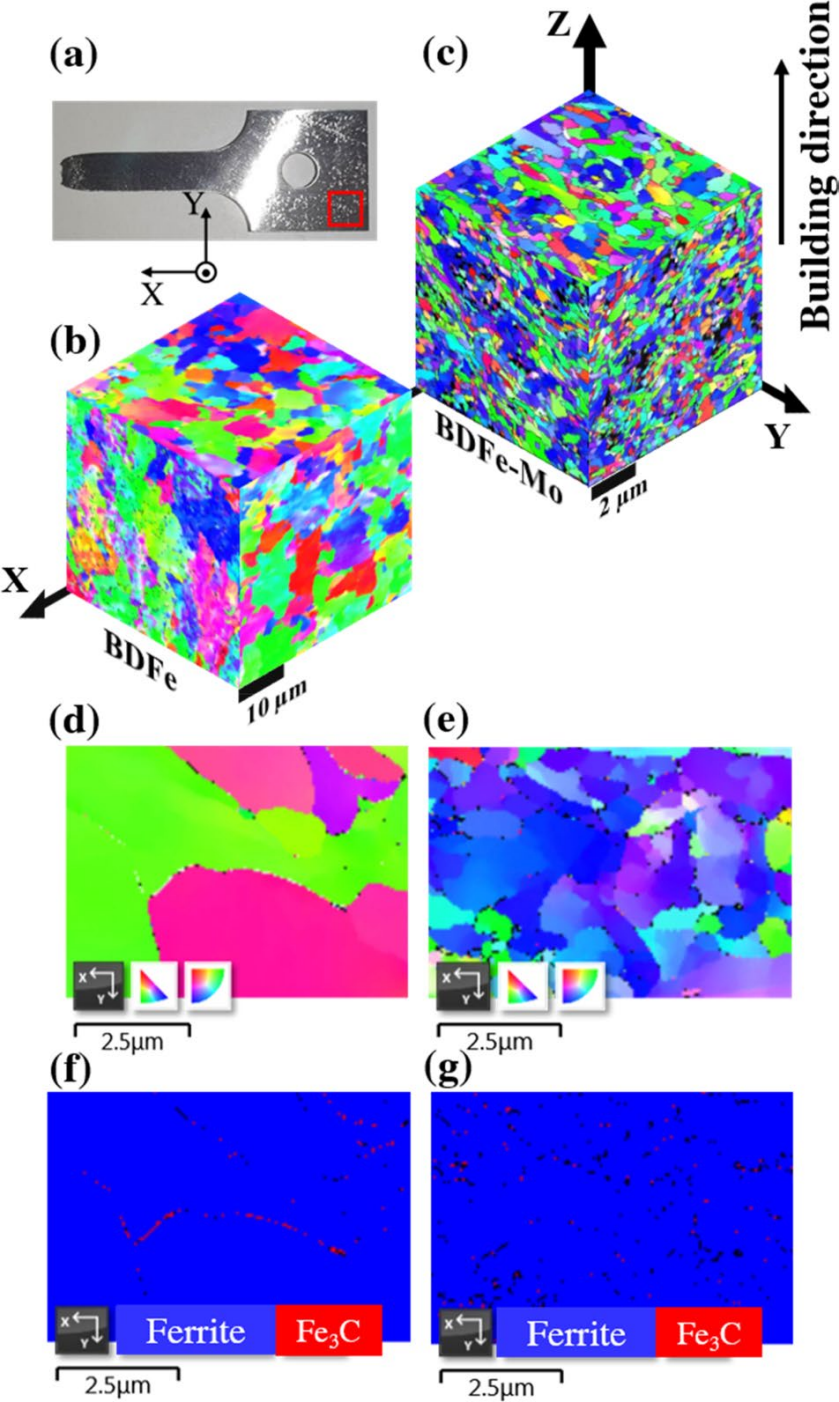
(**a**) Schematic illustration of the position for EBSD measurement. EBSD analysis in the three orthogonal planes in the (**b**) BDFe and (**c**) BDFe-Mo. EBSD images in the XY plane in the (**d**) BDFe and (**e**) BDFe-Mo. Crystal orientation map in the (**f**) BDFe and (**g**) BDFe-Mo specimens.

The microstructural evolution in the three orthogonal planes (XY, YZ, and XZ) was characterized using electron backscatter diffraction (EBSD) to clarify the grain shape, grain size, and second phase precipitation of the two SLM-built BDFe specimens, as described in Fig. 5. EBSD analysis was taken at the corner of the tensile specimens (as illustrated by the red marked square in Fig. 5a), where it is assumed not to be experienced the plastic deformation. Both BDFe samples exhibited a random distribution of majorly equiaxed grains accompanying with some slightly elongated grains, in agreement with similar observations on the grain shape appeared during SLM fabrication process. The building direction in the SLM-built BDFe samples was along the Z-axis, as shown in Fig. 5b,c. The formation of slightly elongated grains was ascribed to temperature gradients in the melted pre-additive manufacturing powder layer along the building direction during rapid solidification process^59–63^.

It can be seen from Fig. 5b,c that the grain size of the BDFe-Mo was much smaller than that of the BDFe. The image*J* software was employed to determine the average grain sizes of the two BDFe samples in the three orthogonal planes, reported in Table 2. The distribution of grain size was not uniform in the three orthogonal planes in which the grain sizes in the XZ and YZ planes were similar but they were smaller than those in the XY plane for both BDFe specimens. To calculate the strength enhancement as a contribution of grain boundary strengthening in the two BDFe samples, the empirical Hall–Petch (H–P) relationship was analyzed based on Eq. (1)^24,64^.

1 $$(\Delta \sigma_{cr} = kD^{-{1/2}})$$

where $\Delta \sigma_{cr}$ is the increment of yield strength, *k* is the H–P slope of the contribution of grain boundary strengthening, and *D* is the average grain size. Following the previously reported work, the value of *k* was chosen as 20 MPa/mm^1/2^^65^. The calculated yield strengths using the average grain sizes of the three orthogonal planes were determined to be 392.19 and 1,105.62 MPa which were slightly smaller than the macroscopic yield strengths of 451 and 1,193 MPa in the BDFe and BDFe-Mo obtained from the S–S curves, respectively.

**Table 2:** The calculated strength enhancements using the average and dominant grain sizes of the three orthogonal planes in the two BDFe samples in comparison with the macroscopic yield strengths obtained from the S–S curves.

	Average grain size (μm)	Strength enhancement (MPa)	Macroscopic yield strength (MPa)	Dominant grain size (μm)	Strength enhancement (MPa)
**BDFe**					
XY plane	3.16	355.78	451	2.09	437.58
XZ plane	2.37	410.82		1.80	472.06
YZ plane	2.38	409.96		1.93	455.25
Average	2.64	392.19		1.94	454.97
**BDFe-Mo**					
XY plane	0.41	987.73	1193	0.33	1102.64
XZ plane	0.29	1174.44		0.25	1254.91
YZ plane	0.30	1154.70		0.26	1237.97
Average	0.33	1105.62		0.28	1198.51

Since there was a noticeable difference of grain size in each plane, the average of dominant grain size may be a more appropriate choice in calculating the strength enhancement. The dominant grain sizes determined by the image*J* revealed smaller values compared with the average grain sizes in both BDFe samples. The calculated yield strengths using the average of dominant grain sizes in the three orthogonal planes were found to be 454.97 and 1198.51 MPa in the BDFe and BDFe-Mo, respectively, indicating a great consistent with the macroscopic yield strengths, as summarized in Table 2. The EBSD results demonstrated the dominant mechanism of grain boundary strengthening for the salient strength enhancement in the BDFe-Mo specimen.

To elucidate the other possible reasons contributing to the strengthening behavior in the SLM-built BDFe specimens, the microstructures of both samples in the XY plane in Fig. 5d,e were coupled with their crystal orientation maps, as shown in Fig. 5f,g in the same magnification. In accordance with the XRD results, both BDFe samples revealed the ferrite grains together with the second phase precipitate at the grain boundaries which was identified as the cementite (Fe_3_C). In the Fe-based alloys, the metal carbides precipitate out of the supersaturated ferrite^66,67^ and form at the grain boundaries^68^. The growth of Fe_3_C precipitates was assigned to the increased C content coupled with the partitioning of Si occurring around 300 °C as the SLM-built specimens experienced the consecutive reheating cycles^69^. The precipitation of Fe_3_C at the grain boundaries caused the pinning effect in inhibiting the growth rate of matrix grains and thus refined the ferrite grains. The existence of more grain boundaries due to the increased contents of C and Si promotes the ferrite nucleation for achieving a uniformly finer grained microstructure in the BDFe-Mo. Moreover, the increased dislocation density during the successive heating and cooling sequences derived from a large difference of CTE between the matrix and Fe_3_C contributed to the strength enhancement^69^. The precipitation strengthening of Fe_3_C at the grain boundary greatly enhanced with the increased C concentration and this strengthening mechanism significantly contributed to the macroscopic yield strength^70^. The results of microstructure investigation indicated that the ferrite grain refinement accompanying with the precipitation strengthening were the major strengthening mechanisms for the superior mechanical strength in the BDFe-Mo.

**Figure 6:**
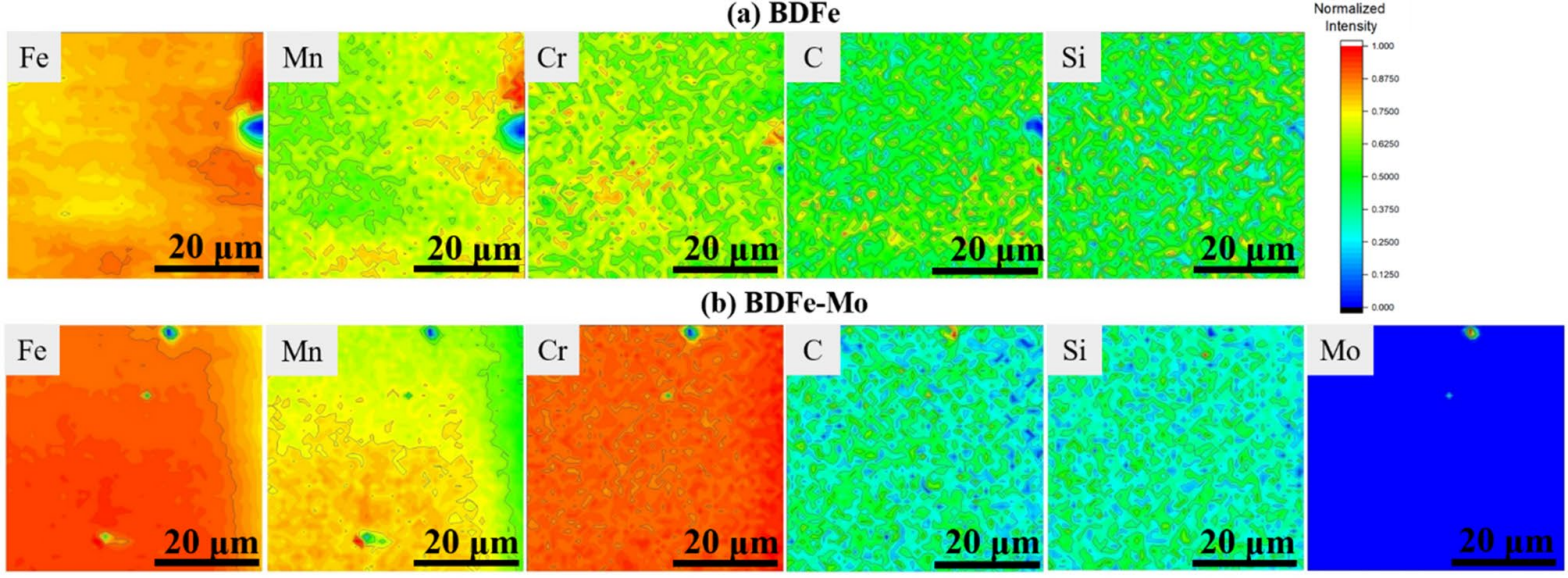
XRF maps of the constituent alloying elements in the (**a**) BDFe and (**b**) BDFe-Mo samples.

Since the primary reason affecting the grain boundary strengthening between the two SLM-built BDFe specimens is the difference in their constituent microalloying elements, an examination towards the chemical distribution of individual alloying elements via X-ray fluorescence (XRF) mapping was necessary. The XRF maps in Fig. 6a exhibited a localized chemical inhomogeneity of the constituent alloying elements where the Fe–Mn, Fe–Cr rich regions, and Cr-C-Si segregation were obtained in the BDFe specimen. The heterogeneous distributions of alloying elements in the BDFe-Mo were likely similar to those in the BDFe in which the Fe–Mn rich region was associated with a more uniform distribution of Cr, C, and Si (Fig. 6b). Furthermore, a preferred segregation of Mo with C was visible in the BDFe-Mo specimen. Low mobility of Mn-induced large undercooling during phase transformation from the austenite to the fully ferrite resulted in strong nucleation of ferrite during cooling in the Fe–Mn alloy^71^. The dissolved Cr in the Fe matrix impeded the dislocation movement and thus strengthened the materials, due to the lattice distortion caused by a large atomic size difference between Fe and Cr^72^. The addition of Mo in the BDFe-Mo has been found to facilitate the solid solution strengthening effect and possibly induce significant ferrite grain refinement due to the delay of dynamic recrystallization^73^. The grain refinement may also occur with the existence of Mo and the increased Mn content, which could lower the temperature for phase transformation from the austenite to the ferrite^74^. Furthermore, the formed Mo-C rich region in the BDFe-Mo may attribute to the precipitation hardening^73^. The impressive mechanical strength behavior in the BDFe-Mo inferred that an appropriate increase in the concentration of microalloying elements gave rise to the grain refinement of the ferrite and precipitation strengthening of metal carbides.

**Figure 7:**
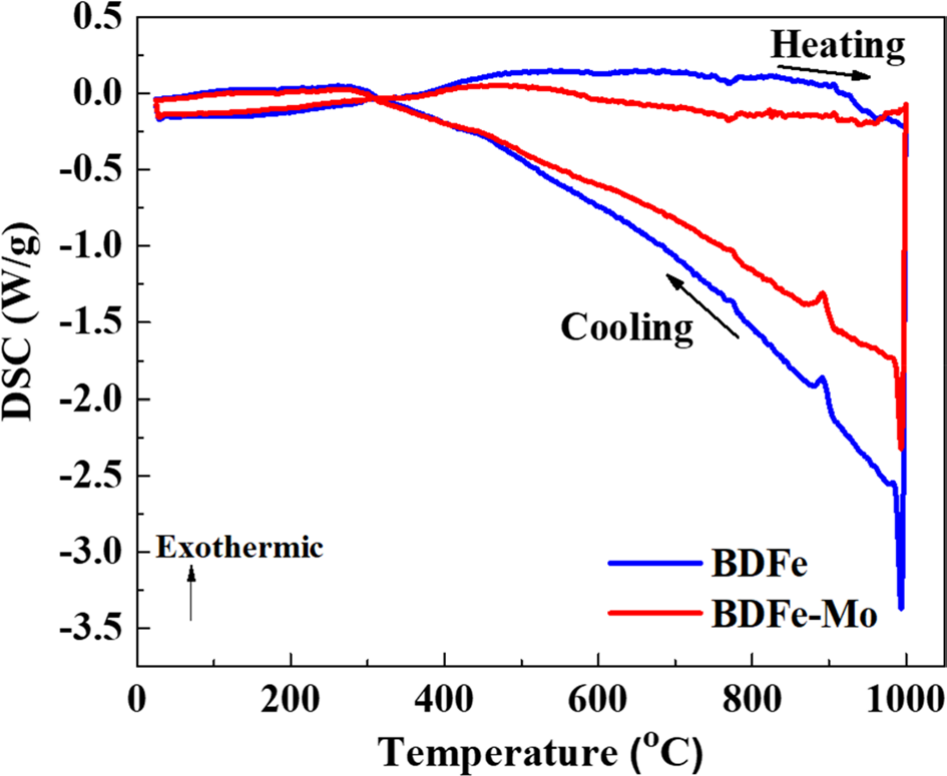
DSC curves of the two BDFe powders during continuous heating and cooling.

In order to better understand thermal behaviors affecting the grain growth during the SLM process, the evolution of heat energy stored in the BDFe powders was explored using differential scanning calorimetry (DSC) analysis. Figure 7 presents the heat absorption and heat release of the BDFe powders during continuous heating and cooling up to 1000 °C. The DSC plots disclosed similar broad exothermic peaks in both BDFe powders upon heating, however, the exothermic peaks were more obvious in the BDFe rather than in the BDFe-Mo. This result indicated more heat energy released in the BDFe and implied more evident grain growth in the BDFe^75^. Similar features were obtained in both BDFe powders upon cooling and the BDFe was more endothermic than the BDFe-Mo, indicating more heat absorbed in the BDFe. The peaks located at 772 °C and 891 °C upon cooling correspond to the Curie temperature and the austenite to ferrite transformation temperature^75^. The ferrite phase with a Fe_3_C observed in the two SLM-built BDFe alloys was beneficial for enhancing high mechanical strength.

## Summary

The SLM-built BDFe alloys with optimal fabrication parameters revealed good mechanical properties. Compared with the BDFe, the BDFe-Mo exhibited remarkably superior YS of 1193 MPa and UTS of 1200 MPa together with a relatively fair elongation of 13.5%. Such a prominent strengthening behavior in the BDFe-Mo was assigned to a significant ferrite grain refinement coupled with the precipitate strengthening mechanism, tailored by the higher optimal concentrations of C, Cr, Mn, and Si accompanying with the addition of Mo. Designing the pre-additive manufacturing microalloying elements with their appropriate contents is promising for additive manufactured parts owning excellent strength-ductility combination without post heat treatments.

## Materials and methods

### Feedstock materials

The two kinds of spherical powders, BDFe with Fe purity higher than 99.5% and BDFe-Mo with Fe purity higher than 98.5%, were used in this study. The elemental compositions of the two powders were examined by X-ray fluorescent method using Rigaku ZSX Primus IV machine and tabulated in Table 3. The two powders were produced by gas atomization method using VIGA equipment technology, which enabled the spherical powder particles to have less amount of interstitial impurities. The particle sizes of the two powders were in the range of 10–60 µm and were determined by air-flow powder classifier.

**Table 3:** Chemical composition of the two BDFe powders.

Sample name	Composition (wt%)					
	Fe	Mn	Cr	C	Si	Mo
BDFe	Bal.	0.12	0.04	0.03	0.01	–
BDFe-Mo	Bal.	0.60	0.20	0.15	0.15	0.12

### Selective laser melting process

A selective laser melting process was carried out using AM100, Industrial Technology Research Institute (ITRI) self-made machine. The working chamber was filled with argon (Ar) gas with outlet pressure of 2 bar in order to maintain the oxygen level below 0.1% for preventing iron oxidation during fabrication process. This machine provides a single laser beam with the spot size of 60 µm and laser power up to 500 W. A S45C steel was used as a base plate with the area of 100 × 100 mm^2^ on the building platform.

Pin-loaded tension test specimens were fabricated in horizontal built orientation using laser power of 150 W, laser speed of 1.2 m/s with the layer thickness of 30 µm. The fabrication process also uses stripe laser scanning strategy with a hatch space of 60 µm. The pin-loaded tension test specimens were prepared with an overall length of 60 mm, gauge length and width of 20 mm and 10 mm, respectively, following ASTM standard E8/E8M-09^76^. For statistical considerations, three pin-loaded tension test specimens of each BDFe powder were measured.

### Electron backscatter diffraction

Microstructure observation via electron backscatter diffraction (EBSD) was performed using the JEOL JSM-6500F/OXFORD Nordlys electron microscope operated at 15 kV and was analyzed using AZTEC software. The undeformed region of the tensile sample was cut into three distinct specimens for the representative microstructures in the three orthogonal planes (XY, YZ, and XZ). The measurement was made at the ambient temperature of 20 °C with a relative humidity of 50%.

### Continuous heating and thermal cycling using temporally coherent X-ray diffraction

*In-situ* synchrotron X-ray diffraction measurements were conducted at Taiwan Photon Source (TPS) 09A beamline (BL) using temporally coherent X-ray diffraction of the National Synchrotron Radiation Research Center (NSRRC). This station was designed for hard X-ray scattering to study the static and dynamic structure behaviors of crystalline materials using a 9-circle diffractometer coupled with a heater, which can heat the two BDFe powders up to 900 °C.

Diffraction profiles were recorded during continuous heating at room temperature, then from 50 °C up to 900 °C with an increment of 50 °C and they were analyzed using the General Structure Analysis Software (GSAS)-II, which is an open source software for determination of crystal structures from different phases and lattice sizes^77^. After fitting the diffraction patterns obtained at different temperatures, the changes of the lattice parameters were calculated to yield the CTE. The average linear CTE was calculated using Eq. (2) ^78^.

2 $$CTE = \frac{1}{{L_{0} }}\frac{{L_{1 - } L_{0} }}{{T_{1} - T_{0} }} = \frac{1}{{L_{0} }}\frac{\Delta L}{{\Delta T}}$$

where L_0_ is the initial lattice constant at room temperature T_o_. ΔL is the change in the lattice constant at a specific temperature and ΔT is the change in the temperature.

For thermal cycling test, the two BDFe powders were heated from RT up to 900 °C and cooled down to RT in the 1st cycle. The temperature was heated up to 700 °C and decreased to RT in the 2nd cycle, followed by an increase of temperature up to 500 °C and then cooled to RT in the 3rd cycle. The heating and cooling rates in the thermal cycling test were 10 °C/min. Diffraction patterns were recorded every 100 °C increment during heating and cooling.

### X-ray nano-diffraction

X-ray nano diffraction (XND) at TPS 21A BL of the NSRRC was exploited to map the X-ray fluorescence intensity of each alloying element with a spatial resolution of 90 nm for the examination of chemical homogeneity in the two BDFe samples. A mapping area of 50 × 50 µm^2^ with a step size of 1.0 µm was recorded and each mapping consisted of 1 × 1 pixels. More details of the related protocols are archived^21,79^.

### Differential scanning calorimetry

To examine the thermal behavior subjected to heating and cooling, the two BDFe powders were measured by differential scanning calorimetry. The experiments were conducted by the continuous heating up to 1000 °C from RT with a heating rate of 10 °C/min, followed by the continuous cooling to RT with a cooling rate of 10 °C/min.

## Data Availability

The data will be made available on request.

## References

[1] Costello, K. & Rimol M. *Gartner Identifies Five Emerging Trends That Will Drive Technology Innovation for the Next Decade*. 2020: STAMFORD, Conn. p. https://www.gartner.com/en/newsroom/press-releases/2020-08-18-gartner-identifies-five-emerging-trends-that-will-drive-technology-innovation-for-the-next-decade.

[2] Middleton JC, Tipton AJ (2000). Synthetic biodegradable polymers as orthopedic devices. Biomaterials.

[3] Gilding DK, Reed AM (1979). Biodegradable polymers for use in surgery—polyglycolic/poly(actic acid) homo- and copolymers: 1. Polymer.

[4] Cha P-R (2013). Biodegradability engineering of biodegradable Mg alloys: Tailoring the electrochemical properties and microstructure of constituent phases. Sci. Rep..

[5] Yang H (2020). Alloying design of biodegradable zinc as promising bone implants for load-bearing applications. Nat. Commun..

[6] Kannan MB (2017). Biocompatibility and biodegradation studies of a commercial zinc alloy for temporary mini-implant applications. Sci. Rep..

[7] Yusop AHM (2015). Controlling the degradation kinetics of porous iron by poly(lactic-co-glycolic acid) infiltration for use as temporary medical implants. Sci. Rep..

[8] Huang T, Zheng Y (2016). Uniform and accelerated degradation of pure iron patterned by Pt disc arrays. Sci. Rep..

[9] Witte F (2007). Biodegradable magnesium scaffolds: part 1: appropriate inflammatory response. J. Biomed. Mater. Res. Part A.

[10] Sezer N (2018). Review of magnesium-based biomaterials and their applications. J. Magn. Alloys.

[11] Peuster M (2006). Long-term biocompatibility of a corrodible peripheral iron stent in the porcine descending aorta. Biomaterials.

[12] Wegener B (2020). Development of a novel biodegradable porous iron-based implant for bone replacement. Sci. Rep..

[13] Colombo A, Karvouni E (2000). Biodegradable stents : "fulfilling the mission and stepping away". Circulation.

[14] Chandra G, Pandey A (2020). Biodegradable bone implants in orthopedic applications: a review. Biocybern. Biomed. Eng..

[15] Peuster M (2001). A novel approach to temporary stenting: degradable cardiovascular stents produced from corrodible metal—results 6–18 months after implantation into New Zealand white rabbits. Heart.

[16] Carluccio D (2020). Challenges and opportunities in the selective laser melting of biodegradable metals for load-bearing bone scaffold applications. Metall. Mater. Trans. A.

[17] Obayi CS (2016). Effect of grain sizes on mechanical properties and biodegradation behavior of pure iron for cardiovascular stent application. Biomatter.

[18] Huang EW (2019). Hardening steels by the generation of transient phase using additive manufacturing. Intermetallics.

[19] Tseng JC (2020). Deformations of Ti-6Al-4V additive-manufacturing-induced isotropic and anisotropic columnar structures: insitu measurements and underlying mechanisms. Addit. Manuf..

[20] Chae H (2021). Unravelling thermal history during additive manufacturing of martensitic stainless steel. J. Alloys Compd..

[21] Tsai P-I (2019). Multi-scale mapping for collagen-regulated mineralization in bone remodeling of additive manufacturing porous implants. Mater. Chem. Phys..

[22] Manakari V, Parande G, Gupta M (2017). Selective laser melting of magnesium and magnesium alloy powders: a review. Metals.

[23] Zhang LC (2016). Review on manufacture by selective laser melting and properties of titanium based materials for biomedical applications. Mater. Technol..

[24] Song B (2014). Microstructure and tensile properties of iron parts fabricated by selective laser melting. Opt. Laser Technol..

[25] Carluccio D (2019). Comparative study of pure iron manufactured by selective laser melting, laser metal deposition, and casting processes. Adv. Eng. Mater..

[26] Calcagnotto M (2010). Orientation gradients and geometrically necessary dislocations in ultrafine grained dual-phase steels studied by 2D and 3D EBSD. Mater. Sci. Eng. Struct. Mater. Prop. Microstruct. Process..

[27] Peng-Heng C, Preban AG (1985). The effect of ferrite grain size and martensite volume fraction on the tensile properties of dual phase steel. Acta Metall..

[28] Jiang Z, Guan Z, Lian J (1995). Effects of microstructural variables on the deformation behaviour of dual-phase steel. Mater. Sci. Eng. A.

[29] Lenka S (2013). Effect of recalescence on microstructure and phase transformation in high carbon steel. Mater. Sci. Technol..

[30] Yokota T, Mateo CG, Bhadeshia HKDH (2004). Formation of nanostructured steels by phase transformation. Scr. Mater..

[31] Bajaj P (2020). Steels in additive manufacturing: a review of their microstructure and properties. Mater. Sci. Eng. A.

[32] Sigel A, Sigel H, Sigel RKO (2013). Interrelations between essential metal ions and human diseases. Metal Ions Life Sci..

[33] Redlich C, Quadbeck P, Thieme M, Kiebackb B (2020). Molybdenum—a biodegradable implant material for structural applications?. Acta Biomater..

[34] Tan JH, Wong WLE, Dalgarno KW (2017). An overview of powder granulometry on feedstock and part performance in the selective laser melting process. Addit. Manuf..

[35] Abd-Elghany K, Bourell DL (2012). Property evaluation of 304L stainless steel fabricated by selective laser melting. Rapid Prototyp. J..

[36] Touloukian YS (1994). Thermophysical Properties Research, Thermal Expansion: Metallic Elements and Alloys.

[37] Hull F (1987). Effect of composition on thermal expansion of alloys used in power generation. J. Mater. Eng..

[38] Aslam I (2019). Thermodynamic and kinetic behavior of low-alloy steels: an atomic level study using an Fe–Mn–Si–C modified embedded atom method (MEAM) potential. Materialia.

[39] Gray D. E. *American Institute of Physics Handbook,* (1972).

[40] Hudok D (1990). Properties and selection: irons, steels, and high-performance alloys. Met. Handbook.

[41] Kozlovskii YM, Stankus SV (2019). The linear thermal expansion coefficient of iron in the temperature range of 130–1180 K. J. Phys. Conf. Ser..

[42] Liu YC, Sommer F, Mittemeijer EJ (2004). Calibration of the differential dilatometric measurement signal upon heating and cooling; thermal expansion of pure iron. Thermochim. Acta.

[43] Denand B (2020). Carbon content evolution in austenite during austenitization studied by in situ synchrotron X-ray diffraction of a hypoeutectoid steel. Materialia.

[44] Armentani E, Esposito R, Sepe R (2007). The effect of thermal properties and weld efficiency on residual stresses in welding. J. Achiev. Mater. Manuf. Eng..

[45] Li C (2018). Residual stress in metal additive manufacturing. Procedia CIRP.

[46] Mercelis P, Kruth JP (2006). Residual stresses in selective laser sintering and selective laser melting. Rapid Prototyp. J..

[47] Yakout M, Elbestawi MA, Veldhuis SC (2018). A study of thermal expansion coefficients and microstructure during selective laser melting of Invar 36 and stainless steel 316L. Addit. Manuf..

[48] Liu S (2013). Oxide scales characterization of micro-alloyed steel at high temperature. J. Mater. Process. Technol..

[49] Chen RY, Yuen WYD (2008). Short-time oxidation behavior of low-carbon, low-silicon steel in air at 850–1,180 °C––I: oxidation kinetics. Oxid. Met..

[50] Chen Y-T (2020). Biodegradation ZK50 magnesium alloy compression screws: mechanical properties, biodegradable characteristics and implant test. J. Orthopaedic Sci..

[51] Li W (2020). In vitro and in vivo studies on ultrafine-grained biodegradable pure Mg, Mg–Ca alloy and Mg–Sr alloy processed by high-pressure torsion. Biomater. Sci..

[52] Sikora-Jasinska M (2017). Synthesis, mechanical properties and corrosion behavior of powder metallurgy processed Fe/Mg2Si composites for biodegradable implant applications. Mater. Sci. Eng. C.

[53] Zumdick NA (2019). Additive manufactured WE43 magnesium: a comparative study of the microstructure and mechanical properties with those of powder extruded and as-cast WE43. Mater. Charact..

[54] Hufenbach J (2020). Effect of selective laser melting on microstructure, mechanical, and corrosion properties of biodegradable FeMnCS for implant applications. Adv. Eng. Mater..

[55] Hermawan H (2008). Iron–manganese: new class of metallic degradable biomaterials prepared by powder metallurgy. Powder Metall..

[56] Yang Y (2018). A combined strategy to enhance the properties of Zn by laser rapid solidification and laser alloying. J. Mech. Behav. Biomed. Mater..

[57] Deng Q (2020). Fabrication of high-strength Mg–Gd–Zn–Zr alloy via selective laser melting. Mater. Charact..

[58] Lejcek P (2019). Selective laser melting of pure iron: Multiscale characterization of hierarchical microstructure. Mater. Charact..

[59] Thijs L (2010). A study of the microstructural evolution during selective laser melting of Ti–6Al–4V. Acta Mater..

[60] Amato KN (2012). Microstructures and mechanical behavior of Inconel 718 fabricated by selective laser melting. Acta Mater..

[61] Guan K (2013). Effects of processing parameters on tensile properties of selective laser melted 304 stainless steel. Mater. Des..

[62] Thijs L (2013). Fine-structured aluminium products with controllable texture by selective laser melting of pre-alloyed AlSi10Mg powder. Acta Mater..

[63] Song B (2012). Fabrication and microstructure characterization of selective laser-melted FeAl intermetallic parts. Surf. Coat. Technol..

[64] Callister DW (2000). Materials Science and Engineering an Introduction.

[65] Harwood, J. *Strengthening Mechanisms in Solids*. in *Metals Park: ASM Seminar*. (1960).

[66] Chen C (2009). Precipitation hardening of high-strength low-alloy steels by nanometer-sized carbides. Mater. Sci. Eng. A.

[67] Baker R, Brandon D, Nutting J (1959). The growth of precipitates. Phil. Mag..

[68] Lee W-B (2002). Carbide precipitation and high-temperature strength of hot-rolled high-strength, low-alloy steels containing Nb and Mo. Metall. Mater. Trans. A..

[69] Rodrigues TA (2020). In-situ strengthening of a high strength low alloy steel during wire and arc additive manufacturing (WAAM). Addit. Manuf..

[70] Ganeev AV (2014). On the nature of high-strength state of carbon steel produced by severe plastic deformation. IOP Conf. Ser. Mater. Sci. Eng..

[71] Krielaart GP, Zwaag S (2013). Kinetics of γ → α phase transformation in Fe–Mn alloys containing low manganese. Mater. Sci. Technol..

[72] Song B (2017). Integral method of preparation and fabrication of metal matrix composite: selective laser melting of in-situ nano/submicro-sized carbides reinforced iron matrix composites. Mater. Sci. Eng. A.

[73] Kostryzhev A (2018). Comparative effect of Mo and Cr on microstructure and mechanical properties in NbV-microalloyed bainitic steels. Metals.

[74] Misra RDK (2013). Ultrahigh strength hot rolled microalloyed steels: microstructural aspects of development. Mater. Sci. Technol..

[75] Sha W (2013). Development of structural steels with re resistant microstructures. Mater. Sci. Technol..

[76] International, A. *ASTM E8/E8M-09 Standard Test Methods for Tension Testing of Metallic Materials*. ASTM, (2011).

[77] Toby BH, Von Dreele RB (2013). GSAS-II: the genesis of a modern open-source all purpose crystallography software package. J. Appl. Crystallogr..

[78] James JD (2001). A review of measurement techniques for the thermal expansion coefficient of metals and alloys at elevated temperatures. Meas. Sci. Technol..

[79] Huang EW (2019). Element effects on high-entropy alloy vacancy and heterogeneous lattice distortion subjected to quasi-equilibrium heating. Sci. Rep..

